# Behavioral state resource selection in invasive wild pigs in the Southeastern United States

**DOI:** 10.1038/s41598-021-86363-3

**Published:** 2021-03-25

**Authors:** Lindsay M. Clontz, Kim M. Pepin, Kurt C. VerCauteren, James C. Beasley

**Affiliations:** 1 Savannah River Ecology Laboratory, Warnell School of Forestry and Natural Resources, University of Georgia, PO Drawer E, Aiken, SC 29802 USA; 2 United States Department of Agriculture, Animal and Plant Health Inspection Service, Wildlife Services, National Wildlife Research Center, 4101 LaPorte Avenue, Fort Collins, CO 80521-2154 USA

**Keywords:** Behavioural ecology, Animal behaviour, Invasive species

## Abstract

Elucidating correlations between wild pig (*Sus scrofa*) behavior and landscape attributes can aid in the advancement of management strategies for controlling populations. Using GPS data from 49 wild pigs in the southeastern U.S., we used hidden Markov models to define movement path characteristics and assign behaviors (e.g., resting, foraging, travelling). We then explored the connection between these behaviors and resource selection for both sexes between two distinct seasons based on forage availability (i.e., low forage, high forage). Females demonstrated a crepuscular activity pattern in the high-forage season and a variable pattern in the low-forage season, while males exhibited nocturnal activity patterns across both seasons. Wild pigs selected for bottomland hardwoods and dense canopy cover in all behavioral states in both seasons. Males selected for diversity in vegetation types while foraging in the low-forage season compared to the high-forage season and demonstrated an increased use of linear anthropogenic features across seasons while traveling. Wild pigs can establish populations and home ranges in an array of landscapes, but our results demonstrate male and female pigs exhibit clear differences in movement behavior and there are key resources associated with common behaviors that can be targeted to improve the efficiency of management programs.

## Introduction

Understanding how animals move throughout landscapes and interact with heterogeneously distributed resources is critical for management of invasive species because this knowledge provides insight regarding how populations persist and expand, and is thus one of the central goals of ecological research^1,2^. Habitat characteristics that meet specific needs for different behavioral states (e.g., resting vs. foraging) of an animal are usually spatially segregated; therefore, investigation of movement patterns and habitat selection at a fine spatial scale can be used to illustrate the asynchrony of the behavioral strategies employed over time^3^. The observed movement patterns that make up an animal’s home range are determined by single movement steps that provide information on the interactions between the individual’s external environment and behavioral state^4,5^. Therefore, this interaction represents an animal’s response to the environment^6^. For example, in heterogeneous landscapes an animal can respond to variable stimuli such as food availability, cover, and water that can change the trajectory of their movement path^6^. These responses are ultimately the result of a continual decision-making trade-offs every animal has to make about the wide range of competing demands to survive and reproduce^3^. Understanding these underlying fine-scale interactions with resources allows managers to predict movements of animals in different landscapes to optimize management planning^7,8^.

Despite the relevance of these fine-scale behavioral questions to conservation and management goals, behavior-specific resource selection is understudied in most species due to the lack of behavioral context associated with animal location data^9^. Animal behaviors, and the driving factors behind these behaviors, are difficult to quantify in the absence of proper data resolution and analytical tools^10^. However, continued advancements in global positioning system (GPS) tracking technologies and behavioral analysis techniques provide the ability to estimate behavioral states using movement path characteristics such as turning angles and step-lengths^11–13^. In particular, hidden Markov models (HMM) allow for the exploration of patterns in movement path characteristics created by underlying behavioral states and estimation of the probabilities of transitioning among the identifiable states^10,14,15^. Thus, the application of HMMs to animal relocation data can uncover physiological or behavioral states of tracked individuals, which in turn can be used in a resource selection analysis to infer resource selection associated with identified behaviors.

In the case of an adaptable generalist like invasive wild pigs (*Sus scrofa*), innovative management is critical given the rapid increase in size and distribution of populations throughout their introduced range. In addition, management is important to mitigate the extensive impacts of this species on anthropogenic and natural systems^16,17^. The correlation between behavior and landscape patterns can inform how unexpected populations emerge in new places and continue to expand their range (e.g., travelling via movement corridors and identifying suitable resources to reside), as well as help identify areas that may act as hotspots for disease transmission (e.g., areas associated with close contact behaviors such as resting and foraging). These are major concerns for wildlife managers since wild pigs have the potential to alter ecosystems across broad spatial scales and have extreme economic impacts^6,16–18^. Like most wild animals, the movement behavior of wild pigs is largely driven by spatio-temporal variation in the distribution of resources throughout the landscape^19,20^. Wild pigs move deliberately, choosing different resource patches depending on their current needs (rest, forage, mates, etc.) relative to what is available. Their movements also depend on whether or not the tradeoff for accessing these resources is energetically reasonable^21–23^. When targeting these resources for specific behaviors or needs, wild pig movements tend to be methodical, as they often consistently use the same trails and interact with the same areas on the landscape^24,25^. These patterns tend to change at a broad scale with food availability and dietary shifts throughout the year^26^; however, there is little to no information regarding how wild pigs change fine-scale resource selection and activity patterns associated with specific behaviors as a result of changing landscape characteristics or food availability. Identifying fine-scale behavioral resource selection and activity patterns of wild pigs can inform more effective and efficient selection and development of site-specific management techniques.

In this study, we estimated population-level resource selection patterns (Second Order)^27^ of wild pigs across two distinct periods (hereafter ‘seasons’) based on food availability (high- and low-forage availability) in the Southeast U.S. We then used HMM’s to distinguish and define movement patterns into associated behavioral states (e.g., resting, foraging, traveling) of wild pigs. Lastly, we evaluated the relationship between behavioral states and resource selection. We tested the hypothesis that wild pigs exhibit differential resource selection patterns depending on their behavioral state (Third Order)^27^ and availability of forage resources. We expected females and males to demonstrate different activity patterns throughout the day (i.e., diel patterns: crepuscular, nocturnal, diurnal) due to differences in reproductive responsibilities (e.g., mate seeking, gestation, farrowing) (Table 1). For example, females may have a more variable activity pattern that coincides with farrowing since they reduce movements during the period when piglets have limited mobility^28^ (Table 1). In addition, we expected associated resource selection with movement behaviors to shift throughout the year based on food availability. Overall, given their association with riparian areas^19,29,30^, we expected behavioral states that aligned with restricted movements (i.e., resting and foraging) to be associated with forested areas proximal to water (i.e., bottomland hardwoods) and areas with greater canopy cover, especially in the warmer and mast (e.g., acorns) producing months (Table 1). In contrast, given the heterogeneous distribution of riparian areas throughout our study site we expected wild pigs would more extensively use upland pines and linear features such as roads while traveling (Table 1). During low-forage months, we expected wild pigs to be more opportunistic foragers leading to more variable patterns of resource selection while foraging (Table 1).

**Table 1:** Hypotheses for activity patterns and resource selection for three behavioral states (i.e., resting, foraging, traveling) for female and male wild pigs (*Sus scrofa*) on the Savannah River Site in South Carolina during two distinct seasons based on forage availability, low-forage availability (January–April) and high-forage availability (May–December).

Sex	Behavior	Season	
		Low-forage	High-forage
Females	Resting	Select for areas associated with water and cover	Select for dense thermal cover/areas associated with water
	Foraging	Select for moist landscapes/variable vegetation associated with subterranean food sources	Select for hardwood habitat associated with soft/hard mast
	Traveling	Select for open habitat/linear features	Select for open habitat/linear features
	Activity Pattern	Variable (Farrowing peak)	Typical Nocturnal/Crepuscular
Males	Resting	Select for areas associated with water and cover	Select for dense thermal cover/areas associated with water
	Foraging	Moist landscapes/variable vegetation associated with subterrean food sources	Hardwood habitat associated with soft/hard mast
	Traveling	open habitat/linear features	Open habitat/linear features
	Activity pattern	Typical nocturnal/crepuscular	Typical nocturnal/crepuscular

## Methods

### Study area

**Figure 1:**
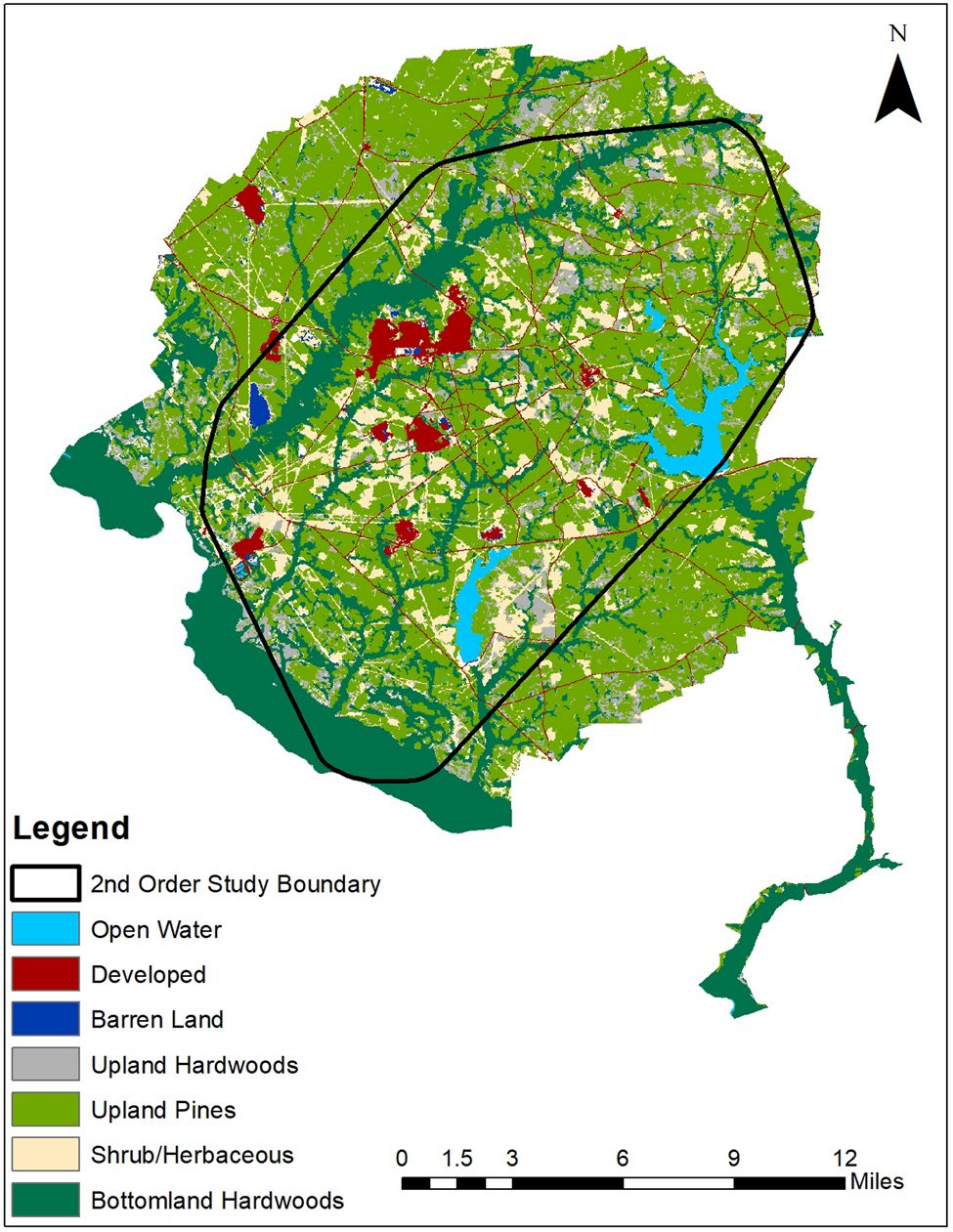
Overall study area with distinct vegetative communities and the 1.2 km^2^ polygon representing the specified area used to develop available locations for second order resource selection functions of male and female wild pigs (*Sus scrofa*) during two distinct seasons (i.e., low-forage and high forage) between Janurary 2014–December 2019 in South Carolina, USA.

Our work was conducted on the Savannah River Site (SRS), a ~ 800 km^2^ site managed by the U.S. Department of Energy (DOE) on the Georgia-South Carolina border (Fig. 1). Although established for industrial activities, facilities and infrastructure comprise a small proportion of the landscape, with most of the landscape being managed by the United States Forest Service (USFS) for timber production and wildlife conservation. The SRS was comprised of approximately 50% upland pine including loblolly pine (*Pinus taeda*), longleaf pine (*Pinus palustris*), and slash pine (*Pinus elliottii*), 25% was bottomland hardwood forest, 10% shrub/herbaceous-dominated areas, 8% upland hardwoods, and the rest was mixed forest, developed, and barren land. Wild pigs have been managed on the SRS since the early 1950s, when an active live-trap-and-removal program was initiated to mitigate damages caused by wild pigs^31^. This program is managed by USFS and currently removes ~ 1300–1800 pigs annually^32^. Despite this control, there are several thousand wild pigs inhabiting the SRS that are distributed throughout the site^33^. Since the SRS was previously used to manufacture nuclear materials and manage nuclear waste^34^, there is limited public access across the site. The diversity of habitat types of the SRS combined with the limited public access, diversity of other wildlife species present, and high wild pig densities (i.e., > 4000–5000 individuals; ~ 5–6.25 individuals/km^2^)^33^ make the site an ideal location to study movement patterns and resource selection of this species.

### Field methods

We captured wild pigs throughout the SRS from January 2014–December 2019 using baited-corral traps equipped with a combination of remote-operated and trip-wire mechanisms. We monitored traps using remote cameras (Reconyx PC900, Holmen, WI, USA) to identify dominant sows to receive GPS collars, as well as all breeding-aged males. We used a dart rifle (X-Caliber, Pneu-Dart Inc., Pennsylvania, USA) to anesthetize captured pigs using a combination of butorphanol [0.077 mg/kg], azaperone [0.026 mg/kg], medetomidine [0.031 mg/kg] (BAM; 0.031 ml/kg; Wildlife Pharmaceuticals Inc., Colorado, USA; Ellis et al. 2019) and Ketamine (2.2 mg/kg; Wildlife Pharmaceuticals Inc., Colorado, USA) or Xylazine (2.2 mg/kg; Wildlife Pharmaceuticals Inc., Colorado, USA) and Telazol (4.4 mg/kg; MWI Veterinary Supply, Idaho, USA). While under anesthesia, we recorded sex and assessed age through examination of tooth eruption^36^. We fit the largest subadult or adult female in each sounder (i.e., social unit) and breeding-aged males (i.e., > 1 year) with an iridium GPS collar (Telonics Gen4 GPS/Iridium System; Sensor weight = 260 g; Total Collar Weight =  ~ 500–880 g, Telonics, Inc., Mesa, Arizona or VECTRONIC GPS PLUS Globalstar-3; Total Collar Weight =  ~ 830 g, VECTRONIC Aerospace, Coralville, Iowa). Anesthetized wild pigs were allowed to recover at the capture site after being reversed with a combination of Atipamezole (25 mg/ml; Wildlife Pharmaceuticals Inc.) and Naltrexone (50 mg/ml; Wildlife Pharmaceuticals Inc., Colorado, USA). Collars were programmed to record GPS locations at 30-min or one-hour intervals and equipped with a mortality sensor that became activated after 12 h with no movement by the animal. To avoid pseudo-replication, we only tracked one individual from each social group and all solitary boars. All experimental protocols were approved by and conducted in compliance with the University of Georgia’s Animal Care and Use Committee (Protocols: A2012 08-004, A2015 05-004, and A2018 08-013), and where applicable, with Animal Research: Reporting In Vivo Experiments (ARRIVE) guidelines.

To estimate location error of GPS transmitters, we left a subset of three collars out for 10 days in fixed locations, 5 days in open vegetation and 5 days in forest vegetation. We used these data to calculate the average error among fixes for each habitat type, and to inform initial parameters for behavioral states.

### Identification of movement states

We used HMMs to model the movement characteristics and associated behavioral states of wild pigs for two distinct seasons based on food availability. We considered January through April to represent a low-food availability time period based on dietary preferences of wild pigs^19^, which also generally represents the peak trapping season in the Southeastern U.S. May through December was considered a high-food availability time period when ample amounts of fruits and plants are available throughout the Spring and Summer months, followed by acorns and other mast in Fall and early Winter. Initially, we used all data collected at 30-min intervals and compared HMM outputs for 30-min locations to outputs of models for the same individuals when subset to 1-h locations, and there were no substantial differences. Therefore, we subset data for wild pigs with a 30-min GPS fix rate to 1-h intervals to maintain an equivalent temporal resolution within our dataset. We also removed any duplicate locations (e.g., same date-time stamp) and locations associated with non-pig movements (e.g., locations after mortality). From collars we were able to retrieve and download, less than 0.01% of locations were 2-Dimensional fixes (i.e., locations collected with three satellites). Therefore, we included all locations regardless of dimensional fix within our dataset to be consistent across all individuals. We also removed the first 48 h of GPS fixes to account for any potential bias associated with residual anesthetic effects.

We used step-lengths and turning angles as our observational input data in HMMs to differentiate among behaviors. We compared HMM results from 25 different sets of randomly chosen starting values for step-lengths and turning angle distribution parameters for each behavioral state to ensure we were capturing global maximums of the likelihood function^12^ (Supplementary Table S1). In addition, using an array of starting values from parameter distributions ensures that models were numerically stable^12^. We tested HMMs with two and three movement states based on model parsimony^13^, but also took into consideration the biological relevance of identified states because model selection criteria sometimes tend to favor models with a greater number of states than makes biological sense^37^.

Sex has been found to be an important predictor of wild pig home range size, with males typically having a larger home range and greater movement rates than females^38^. Also, wild pigs have demonstrated seasonal differences in home range size and habitat selection based on resource availability^19,23,39^. Therefore, we expected sex-specific and seasonal-specific differences in the movement parameters (e.g., step-lengths and turning angles) associated with each behavioral state. We also expected differences in transition probabilities among states throughout the diel period, which ultimately adds to the insight of the model when using it to decode states. We ran two and three movement state HMMs separately for males and females in both the low- and high-forage seasons and tested for an additive effect of time of day on the probability of transitioning among states. Therefore, we ran a total of eight HMMs (Table 2). We selected the most parsimonious model for both seasons for females and males separately using Akaike Information Criterion (AIC)^40^. Next, we decoded the most likely sequence of states to have produced each location in the movement path of each wild pig given the most parsimonious model using the Viterbi algorithm^15^. All computations were done using the moveHMM package^12^ in the statistical computing software R 3.6.1^41^. We partitioned GPS locations into appropriate behavioral states and quantified resource selection for both sexes in each season and behavioral state at the third order (i.e., home range) spatial scale^27^.

**Table 2:** A demonstration of all models ran for female and male wild pigs (*Sus scrofa*) on the Savannah River Site in South Carolina during two distinct seasons based on forage availability, low-forage availability (January–April) and high-forage availability (May–December) separated by type including: (a) hidden Markov models, (b) second order resource selection functions, and (c) third order resource selection functions.

Model	Sex	Season	Covariates
**(a)**			
2-State	Female	Low-forage	Time of day (hour)
2-State	Male	Low-forage	Time of day (hour)
3-State	Female	Low-forage	Time of day (hour)
3-State	Male	Low-forage	Time of day (hour)
2-State	Female	High-forage	Time of day (hour)
2-State	Male	High-forage	Time of day (hour)
3-State	Female	High-forage	Time of day (hour)
3-State	Male	High-forage	Time of day (hour)
**(b)**			
2nd Order RSF	Female	Low-forage	All^a^
2nd Order RSF	Male	Low-forage	All^a^
2nd Order RSF	Female	High-forage	All^a^
2nd Order RSF	Male	High-forage	All^a^
**(c)**			
Resting	Female	Low-forage	All^a^
Foraging	Female	Low-forage	All^a^
Traveling	Female	Low-forage	All^a^
Resting	Male	Low-forage	All^a^
Foraging	Male	Low-forage	All^a^
Traveling	Male	Low-forage	All^a^
Resting	Female	High-forage	All^a^
Foraging	Female	High-forage	All^a^
Traveling	Female	High-forage	All^a^
Resting	Male	High-forage	All^a^
Foraging	Male	High-forage	All^a^
Traveling	Male	High-forage	All^a^

^a^All covariates includes distance to upland pines, distance to upland hardwoods, distance to streams, distance to shrub/herb, distance to secondary road, distance to primary road, distance to bottomland hardwoods, and percent canopy cover.

### Resource selection analyses

#### Habitat covariates

We generated individual raster layers for five types of land cover from the 2016 National Land Cover Database (NLCD) raster layer (30 × 30 m-resolution)^42^ for resource selection analyses: (1) upland pines, (2) bottomland hardwoods, (3) shrub and herbaceous, (4) upland hardwoods, and (5) developed (i.e., buildings/structures). We also characterized the distribution of streams and roads within our study area from existing SRS geospatial layers. We classified primary roads as those that were paved and routinely used for travel by SRS employees, whereas secondary roads were unpaved gravel and/or logging roads. We used the Euclidean distance tool in ArcGIS 10.7.1 (Environmental System Research Institute, Inc., Redlands, CA, USA) to calculate the distance to each of the covariates for used and available locations to provide a less ambiguous approach compared to a classification or categorical-approach^43^ (i.e., A location would receive a “0” for the vegetation type it is observed in). Lastly, we used the NLCD 2016 USFS tree canopy cover raster (30 × 30 m-resolution) to estimate the percent canopy cover.

#### Second order

We selected a 481 km^2^ area within the SRS to represent the study area for this analysis. We generated a minimum convex polygon (MCP) around all GPS locations and buffered it by 1.2 km to account for any long distance movements (Fig. 1)^17,35^. We quantified habitat availability for the population at the second order by systematically sampling the study area (every 3rd pixel, i.e., 90 m; available locations) to ensure the entire area was represented yet maintain a dataset that was computationally manageable, compared to random sampling which may involve uncertainty and not effectively represent the overall landscape^44^. We compared these locations to locations classified as ‘used’ generated by systematically sampling (every 3rd pixel, i.e., 90 m; used locations) within a 95% fixed kernel home range for each individual. Uniformly sampling locations across home ranges allows a comprehensive representation of the resources within a home range to compare to the available locations within the study area. We used the adehabitat package with the reference bandwidth (href) smoothing parameter^45^ in the statistical computing software R 3.6.1^41^ to generate and sample all home ranges. We created individual home ranges for both seasons to compare seasonal shifts in home range distribution. We evaluated used locations specific to each individual home range against the same set of available locations throughout the study area for all individuals. We calculated Pearson’s correlation coefficients to test for collinearity between each of our covariates and excluded covariates with a Pearson’s |r|> 0.6^3^. Covariates that were highly correlated included distance to primary roads and distance to buildings/structures (r = 0.75); therefore, we retained the covariate of distance to primary roads only for modeling purposes given this covariate was more represented in the areas wild pigs were captured. We then fit a global (i.e., including all covariates) generalized linear model (GLM) with binomial response distribution (logistic regression) and logit link to the used-available data individually for both sexes in both the low-forage and high-forage seasons^46,47^. This resulted in four comprehensive models representative of second order resource selection for females and males in the low-forage season and high-forage seasons (Table 2). We standardized all variables prior to model development [(*x*_*i*_ − $\overline{\text{x}}$)/s] (Supplementary Table S2). We then back-transformed, exponentiated, and raised all distance variable coefficients to the one-hundredth power to represent 100 m increments and canopy cover to the tenth power to represent 10 percent increments for interpretation using predictive odds ratios. We did not use a model selection technique to rank candidate models because a global model included the full set of covariates that were of interest for hypothesis testing and, therefore, allowed a direct comparison between coefficient estimates across sexes and seasons^48^. All GLM models were computed using the glm function in R version 3.6.1^41,49^. We assessed how well the second order model explained the data using area under the receiver-operating characteristic curve (AUC^50–52^), which we computed using the pROC package in R version 3.6.1^41,53^. A value of 0.5 indicates the model provides predictions that are no better than random predictions, but values greater than 0.7 indicate a better model fit with more accurate predictions^51^.

#### Third order

To assess fine-scale resource selection of wild pigs, we used a resource selection function (RSF) framework^47^ to compare resource selection of wild pigs across the three behavioral states associated with the movement path characteristics identified from the HMM (i.e., resting, foraging, and traveling). We quantified habitat availability for individuals at the third order by comparing GPS locations (i.e., used locations) to systematically sampled locations (every 3rd pixel, i.e., 90 m; available locations) within home ranges across each of the aforementioned covariates (see above). The sampling framework provided inference on the similarities and differences of wild pig resource selection in three prominent behavioral states that can be extracted to the population level. We used a generalized linear mixed model (GLMM) with binomial response distribution (i.e., used vs. available, logistic regression)^46^, logit link, and a random intercept to account for variation among individuals ^54^. We standardized all variables prior to model development [(*x*_*i*_ − $\overline{\text{x}}$)/s]. We then back-transformed, exponentiated, and raised all distance variable coefficients to the one-hundredth power to represent 100 m increments and canopy cover to the tenth power to represent 10 percent increments for interpretation using predictive odds ratios. All GLMM models were computed using the lme4 package in R version 3.6.1^41,49^.

We calculated Pearson’s correlation coefficients to test for collinearity between each of our covariates^3^. We created a global model including all covariates for each sex in each behavioral state in each season (i.e., 2 sexes × 3 behavioral states × 2 seasons = 12 RSFs) (Table 2). As with our second-order analyses, we did not use a model selection technique, and used AUC to assess how well the model explained the data^50–52^.

### Disclaimer

This manuscript was prepared as an account of work sponsored by an agency of the United States Government. Neither the United States Government nor any agency thereof, nor any of their employees, makes any warranty, express or implied, or assumes any legal liability or responsibility for the accuracy, completeness, or usefulness of any information disclosed, or represents that its use would not infringe privately owned rights. Reference herein to any specific commercial product, process, or service by trade name, trademark, manufacturer, or otherwise does not constitute or imply its endorsement, recommendation, or favoring by the United States Government or any agency thereof. The views and opinions of the authors expressed herein do not necessarily state or reflect those of the United States Government or any agency thereof.

## Results

### Identification of movement states

We used a sample of 49 wild pigs tracked between January 2014 and December 2019, resulting in 117,150 validated and cleaned GPS locations (Table 3). In the low-forage season (January–April), we tracked 37 wild pigs (21 females, 16 males), resulting in 47,983 GPS locations, and in the high-forage season (May–December) we tracked 41 wild pigs (20 females, 21 males), resulting in 69,177 GPS locations (Table 3). From these data, we estimated movement path characteristics (e.g., behavioral states) for 29,433 and 42,277 locations for females during the low- and high-forage seasons, respectively. For males, we had 18,550 locations during the low-forage season and 26,900 during the high-forage season to inform our analyses (Table 3). We determined average collar error in forested vegetation to be 22.3 m and in open vegetation to be 11.9 m.

**Table 3:** Summary of global positioning system (GPS) information, average step-lengths (± SE of the mean parameter) and turning angles of female and male wild pigs (*Sus scrofa*) on the Savannah River Site in South Carolina based on GPS locations from January 2014–December 2019.

Sex	Months (season)	Number of pigs	Number of locations	Mean number ± SE of locations	Range of locations per individual	Avg. step-length ± SE (m)	Avg. turning angle (radians)
Females	January–April	21	29,433	1401.57 ± 137.97	240–2987	124.32 ± 1.23	1.72 ± 0.006
	May–December	20	42,277	2113.85 ± 360.46	432–5843	144.97 ± 1.06	1.68 ± 0.005
Males	January–April	16	18,550	1159.38 ± 174.59	328–2232	186.01 ± 2.36	1.67 ± 0.007
	May–December	21	26,900	1280.95 ± 276.67	239–4263	229.18 ± 2.31	1.62 ± 0.006

**Figure 2:**
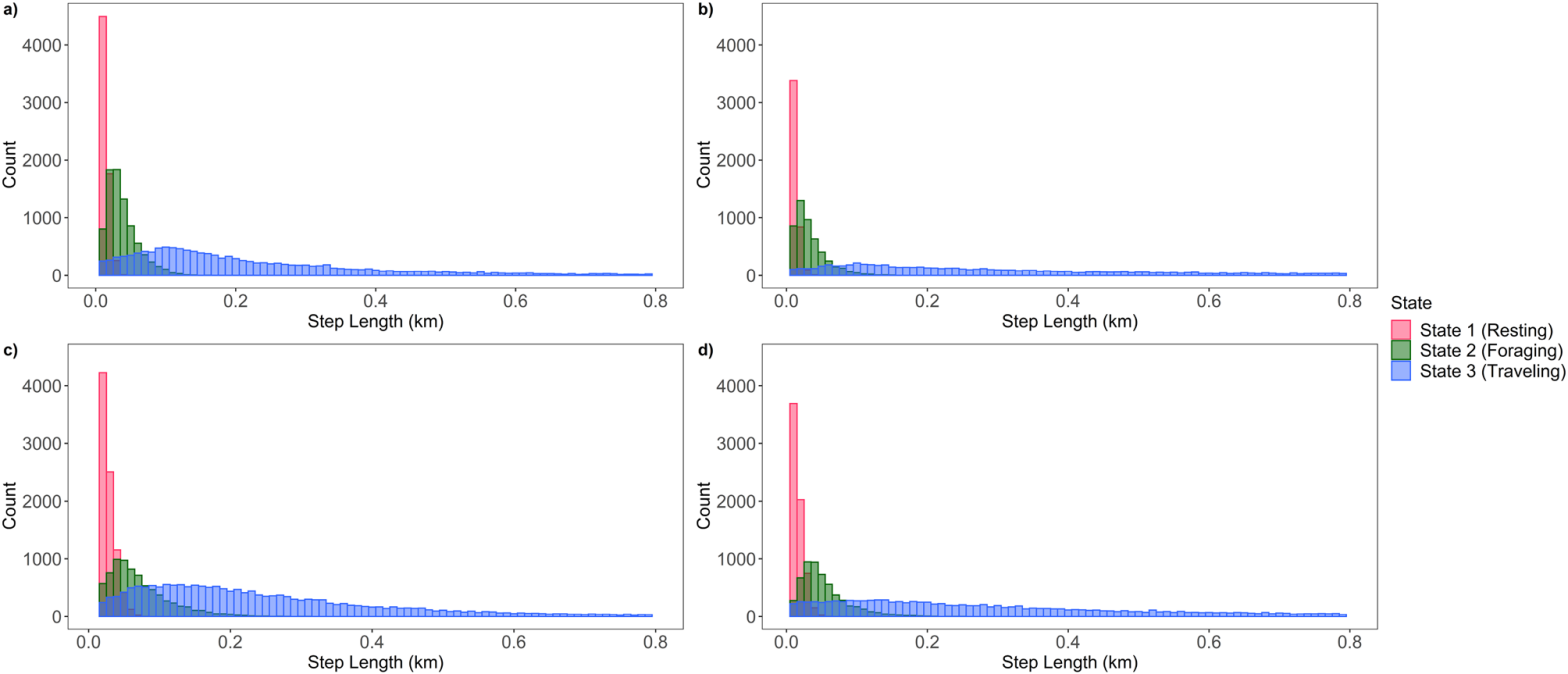
Step-length parameter distributions from three-state hidden Markov models (HMMs) for wild pigs (*Sus scrofa*) in the Southeast USA by sex and season: (**a**) females in low-forage months (January–April); (**b**) males in low-forage months; (**c**) females in high-forage months (May–December); (**d**) males in high-forage months.

**Figure 3:**
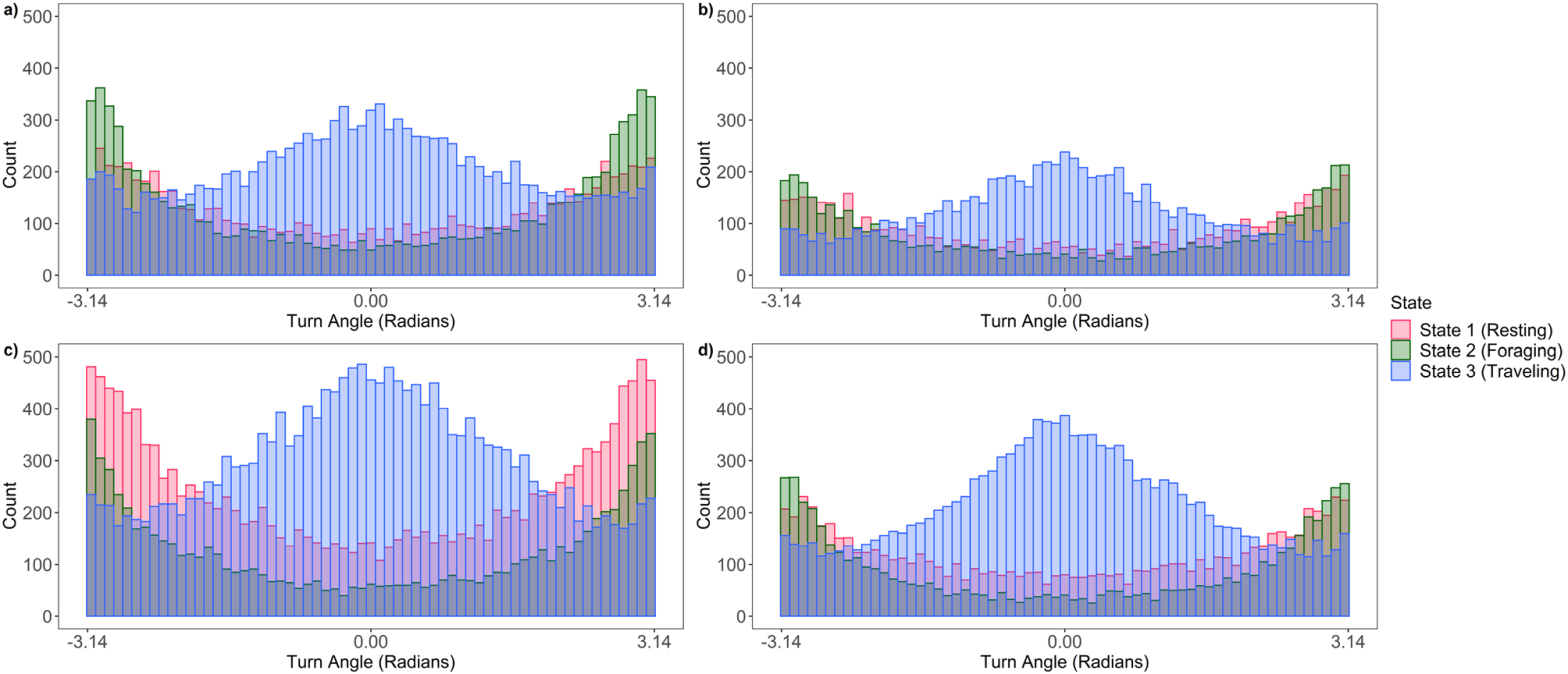
Turn angle parameter distributions from three-state hidden Markov models (HMMs) for wild pigs (*Sus scrofa*) in the Southeast USA by sex and season: (**a**) females in low-forage months (January–April); (**b**) males in low-forage months; (**c**) females in high-forage months (May–December); (**d**) males in high-forage months.

We concluded a three-state HMM with a Gamma distribution for step-length, a wrapped Cauchy distribution for turning angle, and an added covariate of hour in the diel period fit the data of both sexes in both seasons best and provided the most reasonable biological interpretation (Supplementary Table S3). From the three-state HMMs, we identified three general types of movements associated with common behavioral states: (1) a state with short step-lengths and high degrees of turning concentrated around π radians; (2) a state with short to intermediate step-lengths and high degrees of turning concentrated around π radians; and (3) a state with long step-lengths and more straightforward movements with turning concentrated around 0 radians, which likely represents resting, foraging, and traveling behaviors, respectively (Table 4; Figs. 2, 3).

**Table 4:** Average step-lengths (± SE) and turning angles for each designated behavioral state by sex in the 3-state HMMs with the additive effect of hour of day of wild pigs (*Sus scrofa*) on the Savannah River Site in South Carolina based on GPS locations from two distinct seasons based on forage availability, low-forage availability (January–April) and high-forage availability (May–December).

	January–April			May–December		
	Resting	Foraging	Traveling	Resting	Foraging	Traveling
**Mean parameters—females**						
Average step-lengths ± SE (m)	11.4 ± 7.38	37.70 ± 23.24	244.30 ± 220.97	19.25 ± 13.08	67.29 ± 48.32	276.62 ± 227.91
Average turn angle (radians)	3.14	3.11	0.001	− 3.11	3.14	0.07
**Mean parameters—males**						
Average step-lengths ± SE (m)	9.68 ± 6.31	33.00 ± 23.11	398.43 ± 385.81	14.27 ± 9.56	52.46 ± 34.67	420.70 ± 406.12
Average turn angle (radians)	− 3.12	− 3.12	− 0.04	3.11	3.13	0.02

**Figure 4:**
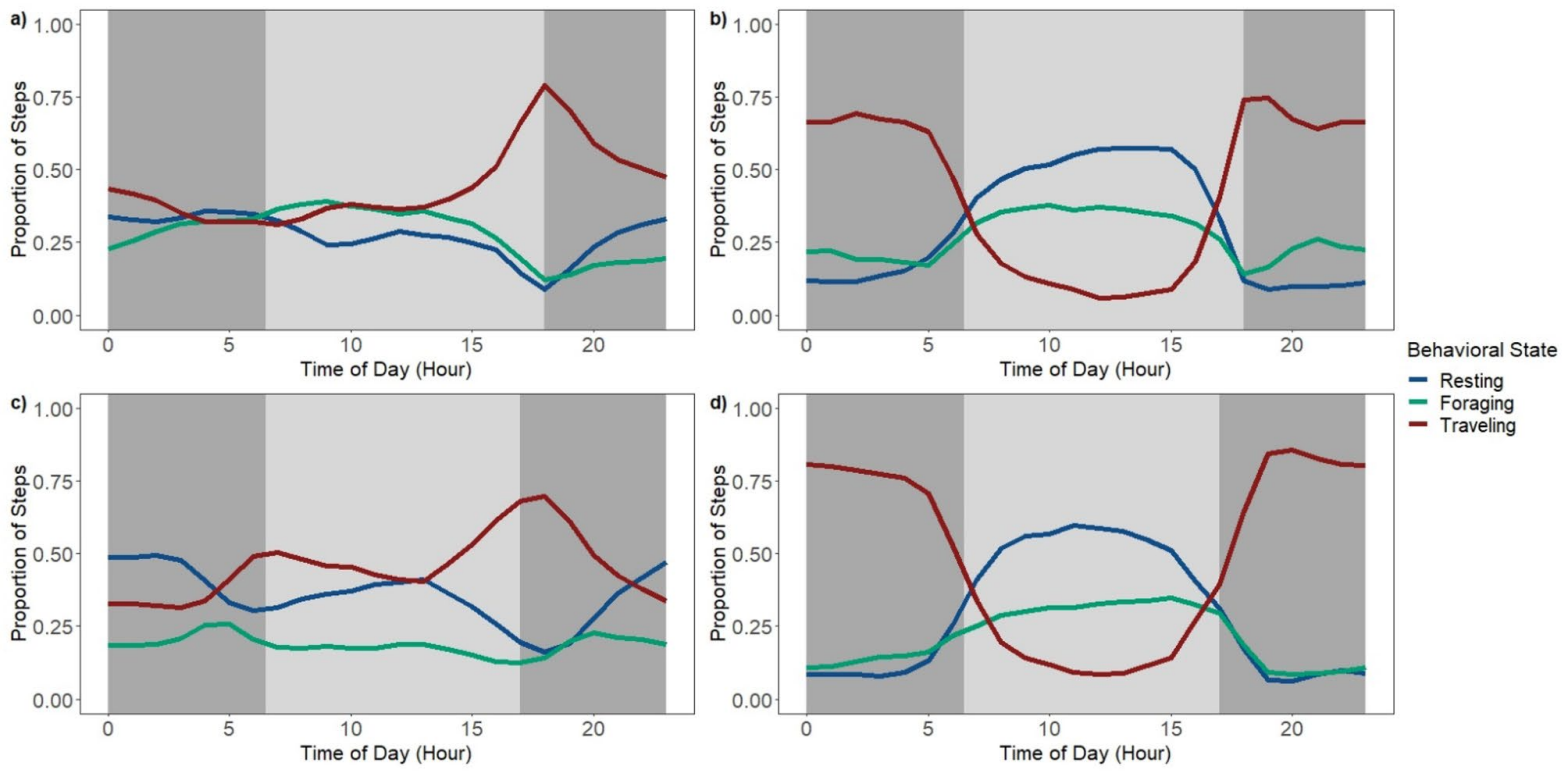
Proportion of steps per hour for each behavioral state of wild pigs (*Sus scrofa*) on the Savannah River Site in South Carolina by sex and season: (**a**) females in low-forage months (January–April); (**b**) males in low-forage months; (**c**) females in high-forage months (May–December); (**d**) males in high-forage months. The dark gray bars represent average nighttime hours while the light gray bar represents the average daytime hours.

Male and female wild pigs exhibited clear differences in movement behavior. Specifically, average step-lengths differed between sexes, and males and females exhibited differences in partitioning of behavioral states across the diel period (Fig. 4). Males typically traveled farther than females in hour segments (Table 3) and demonstrated evident nocturnal activity by traveling mainly throughout the nighttime hours and resting during most of the day (Fig. 4). Males also maintained a consistent movement pattern across seasons. In contrast, females exhibited their longest step-lengths in the evening hours around dusk in the low-forage season and had a variable behavioral pattern throughout the remainder of the day. However, in high-forage months females had a crepuscular activity pattern with peak traveling and foraging movements around dawn and dusk (Fig. 4). Step-lengths for both sexes were longer during the resting and foraging behaviors in the high-forage season compared to the low-forage season (Table 4).

### Resource selection

#### Second order

**Figure 5:**
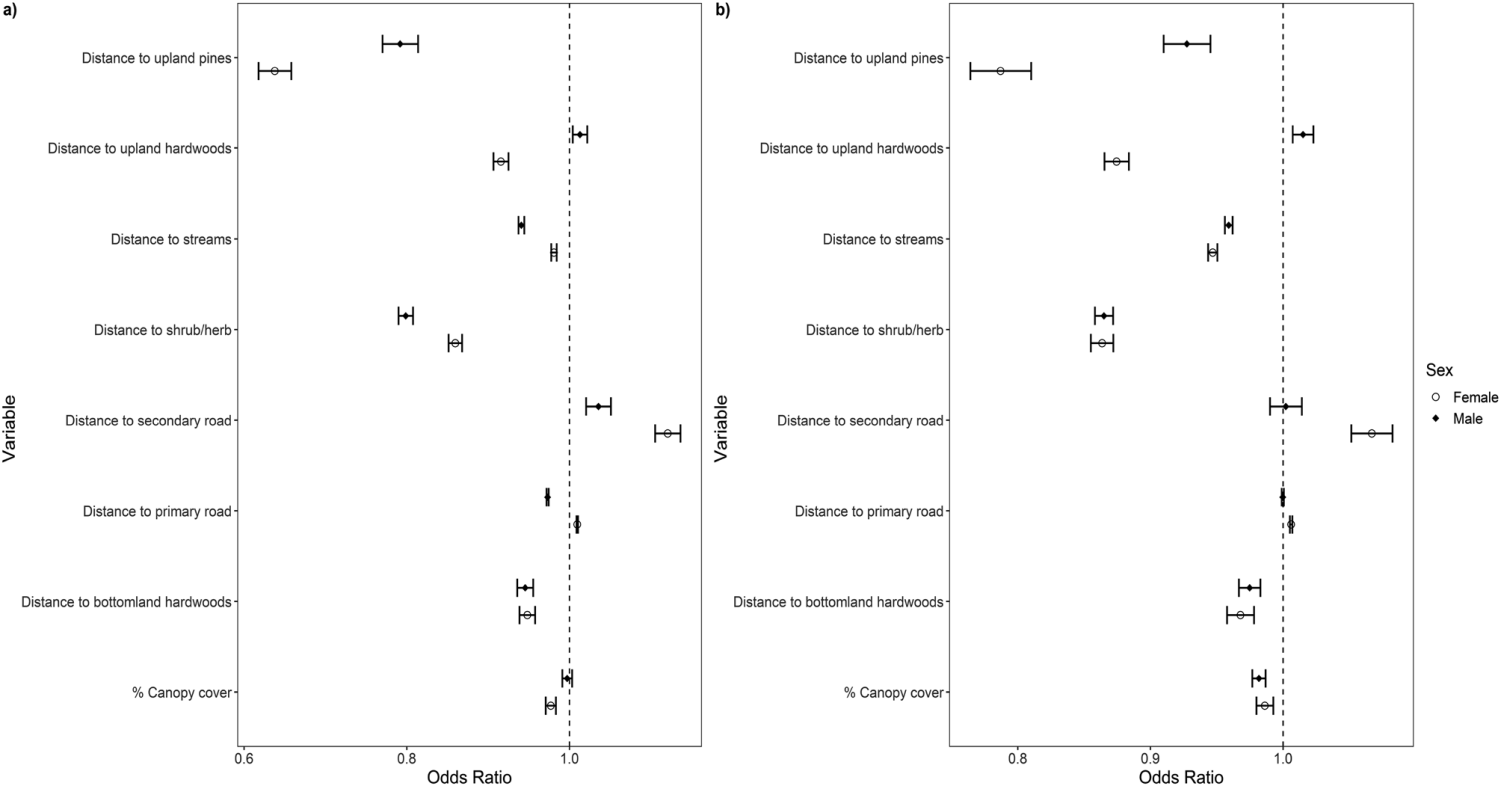
Predictive odds with 95% confidence intervals for second order selection (Johnson 1980) of female and male wild pigs (*Sus scrofa*) on the Savannah River Site in South Carolina during two distinct seasons based on forage availability, (**a**) low-forage availability (January–April) and (**b**) high-forage availability (May–December), for every 100 m increase for distance variables and every 10% increase for canopy cover. In cases where the confidence interval crosses 1, the variable is considered not significant.

Female wild pigs selected all vegetation types (i.e., upland pines, upland hardwoods, bottomland hardwoods, shrub/herbaceous) across our study area in their home-range placement at the second order in both the low and high-forage seasons (Fig. 5, Supplementary Table S4), likely reflecting the ubiquitous establishment of wild pigs across the Savannah River Site^33^. Females also selected locations closer to streams and avoided areas near roads. In contrast, males in the low-forage season selected home ranges in or near upland pines, shrub/herbaceous vegetation, and bottomland hardwoods (Fig. 5). In addition, males selected areas close to streams and primary roads. During the high-forage season, males selected resources similarly to the low-forage season, with the main difference of primary roads no longer being an important driver of home range placement (Fig. 5). AUC values in the low-forage season models for females and males were 0.62, 0.66 and in the high-forage season as 0.64, 0.59, respectively.

#### Third order

**Figure 6:**
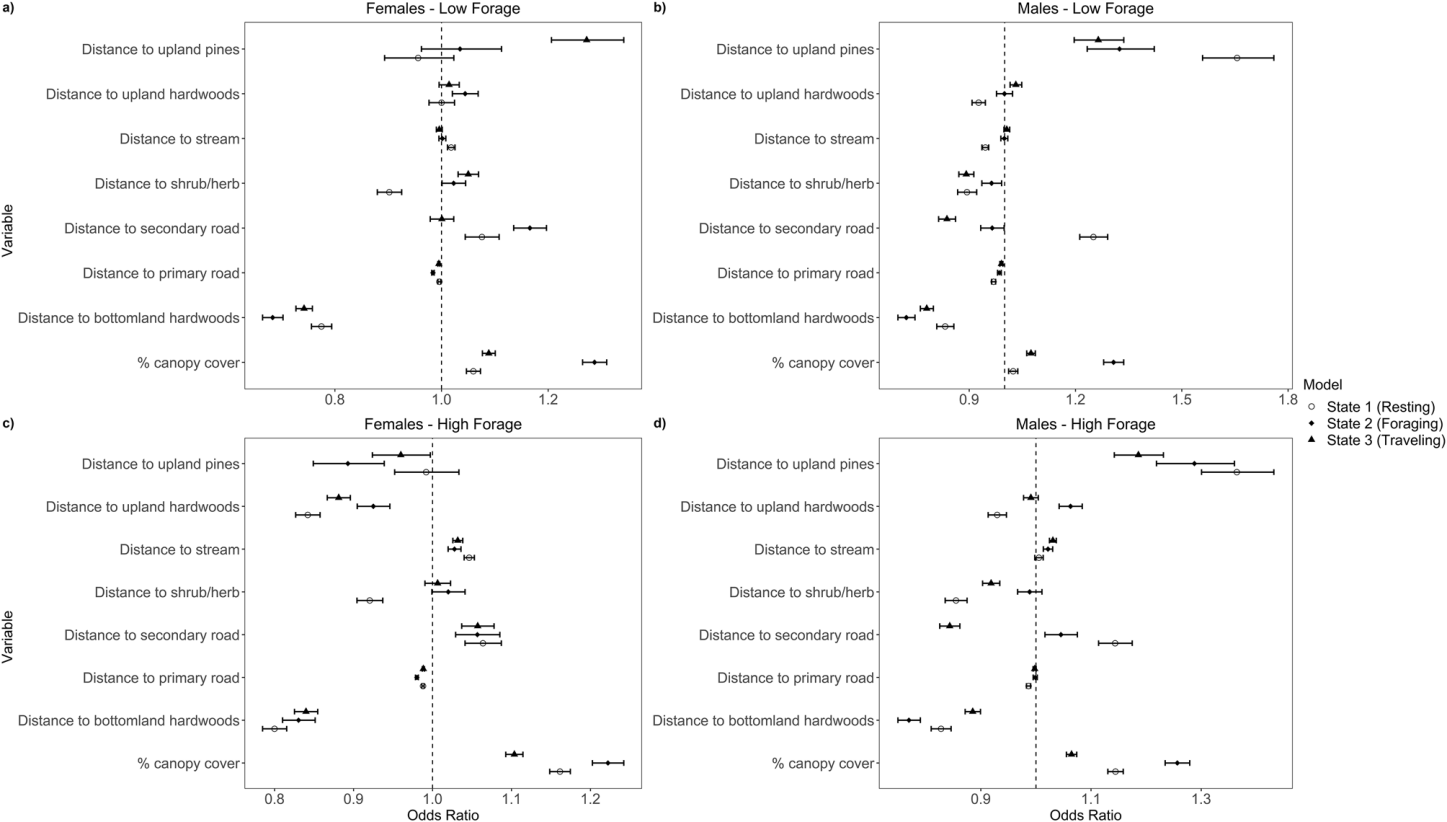
Predictive odds with 95% confidence intervals of third-order selection of male and female wild pigs (*Sus scrofa*) on the Savannah River Site in South Carolina during two distinct seasons based on forage availability [i.e., low-forage season (January–April) and the high-forage season (May–December)]. It demonstrates selection or avoidance of vegetation types, streams, and characteristics of development (e.g., roads) for every 100 m increase and canopy cover for every 10% increase by state where states represent resting, foraging, and traveling behaviors, respectively: (**a**) Females in low-forage months (January–April); (**b**) males in low-forage months; (**c**) females in high-forage months (May–December); (**d**) males in high-forage months. In cases where the confidence interval crosses 1, the variable is considered not significant.

During the resting state, female wild pigs in the low-forage season strongly selected areas in or close to bottomland hardwoods and shrub/herbaceous habitats (Fig. 6, Supplementary Table S5). For example, there was a 23% decrease in use for every 100 m farther away from bottomland hardwoods, and a there was a 10% decrease in use for every 100 m farther away from shrub and herbaceous habitats. During the high-forage season, female wild pigs selected resting areas similarly to the low-forage season with the addition of a strong selection for upland hardwoods (Supplementary Table S5). Also, the resting model for females in both seasons indicated they avoided areas near secondary roads and streams (Fig. 6). Similarly, males selected resting areas in or close to bottomland hardwoods, upland hardwoods, and shrub/herbaceous communities in both seasons. However, males differed between seasons in selecting to rest near streams during the low-forage season but not during the high-forage season. For example, males demonstrated a 5.4% decrease in use for every 100 m farther away from a stream during the low-forage season (Fig. 6).

Throughout the foraging state, females differed in relative probability of selection for specific vegetation types and landscape characteristics between the low- and high-forage seasons (Fig. 6). For example, females selected areas near primary roads and bottomland hardwoods during the low-forage season, yet during the high-forage season they selected areas near upland hardwoods, upland pines, bottomland hardwoods, and areas near primary roads. Males demonstrated more diversity in selection while foraging in the low-forage season including shrub/herbaceous, bottomland hardwoods, and both secondary and primary roads; however, during the high-forage season, males concentrated foraging in areas near or in bottomland hardwood vegetation (Fig. 6). During the high-forage season, males exhibited a 23% decrease in use for every 100 m farther from bottomland hardwoods. In addition, the selection for areas with a high percentage of canopy cover was consistent between sexes and seasons within the foraging behavioral state (Fig. 6).

When traveling, resource selection was similar between seasons for females and males. Females selected primary roads and bottomland hardwoods when traveling in both seasons, with the addition of upland hardwoods in the high-forage season (Fig. 6). Males selected shrub/herbaceous vegetation, primary and secondary roads, and bottomland hardwoods while traveling in both seasons (Fig. 6). For example, in the high-forage season, males displayed a 16% decrease in use for every 100 m farther from secondary roads while traveling (Fig. 6).

The AUC values (overall fit for resting, foraging, and traveling behavioral states in low- and high-forage seasons) were 0.81, 0.79, 0.76 and 0.73, 0.75, 0.73 for females and 0.77, 0.80, 0.70 and 0.77, 0.80, 0.74 for males, respectively.

## Discussion

Wild pigs are a major agricultural and environmental pest in their invasive range, and managing impacts is often expensive and difficult to implement^17^. Therefore, acquiring and analyzing movement data at a fine scale provides important insight on when and where damage or disease transmission is likely to occur. This information provides the ability to improve the efficiency and effectiveness of current management strategies. Therefore, using an extensive dataset of wild pig GPS data across a heterogeneous landscape in the Southeastern U.S., here we demonstrate the differential resource selection tactics employed by wild pigs at both broad (i.e., home range placement) and fine (i.e., within-home-range, behavior-specific) spatial scales for males and females across two distinct seasons. Movement path characteristics of wild pigs in our study were influenced by a combination of local and landscape-level habitat attributes such as bottomland and upland hardwoods, streams, secondary roads, and shrub/herbaceous vegetation communities. While males and females tended to select areas to establish home ranges (population scale) similarly, we found notable differences in the fine-scale use of habitats within home ranges between sexes and seasons. However, both males and females selected bottomland hardwood habitats and areas with extensive canopy cover extensively. Further, through the use of step-lengths and turn angles to define behavioral-based resource selection patterns, we found that females and males differed in daily movement patterns. In addition, we found that wild pigs exhibited differential selection of landscape attributes among behavioral states.

Based on the results of our HMM analyses, we distinguished three biologically relevant behavioral states generally based on patterns in the movement characteristics of wild pigs (i.e., resting, foraging, traveling). Previous studies have identified similar patterns for other species^11,37,55^; however, behavioral states associated with movement characteristics may be assigned differently depending on prior knowledge of different animal species and fix rate at which GPS data were collected. Specifically, the interpretation of a behavioral state associated with short to intermediate step-lengths (what we defined as foraging) may differ among species. For example, this category of behavior was defined as “locally active at the kill site” for wolves^56^, “moderately active” for Florida panthers^57^, and “encamped” for American black bears^55^. However, for caribou, this intermediate behavioral state was assigned as “foraging” and was associated with a foraging behavior for black bears as well^11,55^. Although wild pigs exhibit several behaviors that correspond to short and intermediate step-lengths and tight turn angles (e.g., resting, wallowing, rubbing, tusking, foraging, etc.), for management purposes of wild pigs classifying behaviors into resting, foraging, and traveling encapsulated the most common and consistent motivations of space use (e.g., forage, cover, thermoregulation)^19^ as demonstrated by Blasetti et al.^58^ using captive adult wild pigs that spent approximately 58.9% of their time resting, 14% of their time foraging, and 27.1% of their time traveling. Also, wild pigs have demonstrated variable activity patterns that can shift throughout the year^29,59,60^. Classifying these dominant behaviors and understanding that other similar movement-type behaviors are encompassed as well allows the development of knowledge about where to target certain management strategies or further research.

Both females and males decreased movements or traveling behavior in the mid-day, most likely due to the association with high temperatures in the southeast during the high-forage season^19,38^, and males maintained a consistent nocturnal activity pattern between seasons. However, females exhibited seasonal differences in movement patterns that were likely related to reproductive stages of the reproductive cycle throughout the year, as the timing of farrowing is related to the seasonal availability of forage^28,61^. In the low-forage season, which corresponded with peak farrowing in our study area^28^ (Chinn, unpublished data), females demonstrated a sharp increase in traveling at dusk, an increase in foraging throughout daytime hours, a slight increase in resting mid-day, and a distinct increase in resting throughout nighttime hours. However, during the high-forage season when farrowing rates are lower and juvenile pigs are more mobile, females demonstrated a more crepuscular activity pattern compared to the low-forage season. Pre-parturition and parturition-associated behaviors in some wildlife species, such as wild pigs, are associated with reduced movements and home range sizes^26,38^. Irregular and/or reduced movements can continue after parturition causing an unusual activity pattern in females^61^, as we found throughout the low-forage season. While reproduction can make it more difficult to assign behaviors and demonstrate consistent patterns in movements for females, this demonstration of a change in activity patterns across seasons is consistent with previous literature and reveals the rigor of the methods used in this study. Males and females have different reproductive tendencies and responsibilities as a polygamous species^62^ in which males breed multiple females and provide no parental care. Therefore, behavioral differences between sexes likely reflect different reproductive obligations^61^ and should be a focus for further research, as well as a consideration when designing management plans. Also, the overlap in model parameters between the resting and foraging states for males throughout both seasons and females in the low-forage season indicates that these two states may not be distinct throughout parts of the year. Additional information on animal movement through the use of accelerometers or direct observation, for example, would help to differentiate states with similar distributions of step-lengths and turning angles^13,63^.

Although wild pigs are an invasive habitat generalist, our approach of evaluating population-scale resource selection in contrast to fine-scale behavioral resource selection revealed wild pigs exhibit differential selection of habitats relative to spatial scale. In areas where wild pigs are abundant, they often occur throughout the landscape, which was reflected in our second order (i.e., home range placement) analysis as wild pigs established home ranges in areas proximal to streams containing broad availability of most vegetation types present on the landscape. However, although wild pigs are well documented to select for areas near streams^19,32,64^, here we demonstrate this selection is scale dependent, as neither males or females exhibited focused activity within their home ranges around streams across behavioral states. This difference in selection between spatial scales should be considered when targeting an invasive species for management purposes. The second order models for males and females did not demonstrate much strength in the AUC evaluation (< 0.7); therefore, indicating these models do not fit the data exceptionally well. However, we believe this is due to extensive variation in habitat selection among individuals stemming from the fact that wild pigs are a habitat generalist at the population scale.

Wild pigs can demonstrate multiple behaviors in similar vegetation types^65^, but there are certain habitat characteristics and vegetation types that facilitate specific behaviors (e.g., relocation using roads)^6^. Although wild pigs are ecological generalists, they exhibit spatio-temporal differences in resource selection that reflect underlying biological needs (e.g., thermoregulation)^19,39^. Dense cover and areas proximal to water (i.e., bottomland hardwoods) are two key vegetation characteristics that provide resources that pigs require^19^, and we found that females and males selected for bottomland hardwoods and areas with high percentages of canopy cover in every behavioral state during the low-forage season. In addition, wild pigs forage on subterranean foods such as roots and tubers when other sources are scarce^19,66,67^; therefore, selecting bottomland hardwoods and areas with extensive canopy cover typically coincide with these forage types and provide access to water and cover.

While foraging, males selected for a variety of vegetation types and structures throughout the low-forage season. For example, at the home-range scale males demonstrated a change in selection for primary roads between seasons. In the low-forage season, males selected for areas closer to primary roads in all three behavioral states. Also, males selected for secondary roads in the foraging and traveling states at the home-range scale. The selection for areas near or along both primary and secondary roads while foraging is likely due to the decrease in resources in adjacent natural areas and the consistent availability of grasses along open roadsides during the low-forage season^67,68^. These results coincide with the increase in use of urbanized and anthropogenic areas when natural forage is scarce^69,70^. However, the result of wild pigs utilizing roads could shift in other areas that are associated with hunting or shooting pigs on roads. Wild pigs on the SRS are rarely persecuted (i.e., dog hunting, etc.) on roads; therefore, we expect roads are not associated with negative interactions with humans. Lastly, during the resting state females demonstrated selection for shrub and herbaceous vegetation, which was characterized by a mixture of areas in early successional stages and grasslands that both typically occurred together near linear features such as secondary roads, power lines, and streams, while males selected for this vegetation type in every behavioral state. Areas dominated by this vegetation type most likely provided forage, cover, and easy access to linear features when transitioning to traveling in the low-forage season. Therefore, interactions between wild pig behavior and the attributes of vegetation demonstrated in shrub and herbaceous communities in this study allows for the design of a more informed management plan.

During the high-forage season, at the home-range scale males selected for areas closer to secondary roads while traveling but avoided these areas when foraging and resting. Selecting for anthropogenic and natural linear features can help increase an animal’s pace (step-length) and directional movement, which can assist in traversing the landscape quickly when dispersing, searching for a mate, or transitioning between resting and foraging behaviors^19,71,72^. Also, males selected primarily for bottomland hardwoods while foraging in the high-forage season, and females selected for upland and bottomland hardwoods during all behavioral states, likely reflecting the availability of food, water, and cover in these habitats^19^. Selection for bottomland hardwoods is most likely associated with mast producing hardwoods (e.g., oak acorns) and productive plants in the understory throughout summer months, as well as dense cover and proximity to water. Lastly, throughout the high-forage season, males and females avoided streams at the home-range scale, which is likely due to the extensive stream system throughout the SRS and the ability to access dense cover away from streams during times of extreme temperatures. Other studies have demonstrated the insignificance of streams at the home-range scale throughout certain times of the year when water is generally present throughout the landscape^72^. Unlike the second order models, the AUC values of all third order resource selection models were greater than 0.7 indicating good model fits with meaningful predictions.

Wild pigs exhibit substantive behavioral plasticity making them the perfect invasive species^17^. They can adjust their life history strategies such as daily activity patterns to decrease interaction with humans in populated areas. In addition, wild pigs can adjust their diet throughout the year and in a variety of climatic conditions to benefit their long-term survival depending on local environmental conditions^19,69,73,74^. Although our study was limited to the SRS in the Southeastern U.S., wild pigs demonstrate consistent selection patterns for vegetation types associated with certain resources (i.e., water, mast, etc.)^19,26,75–77^. Therefore, our findings are likely applicable in similar areas throughout this species’ native and introduced range. Further research, though, should focus on wild pig behavioral state resource selection in other geographic regions to elucidate spatio-temporal differences in wild pig behavior across areas of differing climate and resource base. In addition, due to rapid growth in body weights and associated limitations of collecting long-term GPS data on free-ranging wild pigs, not all individuals within our dataset were represented across both seasons. We recognize comparing different individuals across seasons could influence the overall results but given our robust sample size, any differences due to individual variation likely would be minor and not alter the ultimate management implications of this work.

While our general findings are consistent with previous literature on wild pig habitat selection, through the investigation of fine-scale movement patterns coupled with behavioral-based resource selection we were able to demonstrate pigs exhibit clear differences in temporal patterns of activity and selection of habitats among behavioral states. Thus, delineating GPS observational data into unique behavioral states provides unique insights into the relative importance of environmental attributes critical to the invasion of an ecosystem or management of a species that may otherwise be obscured through more coarse-scale resource selection approaches^3^.

Accounting for behavior when studying habitat selection can provide more useful and accurate information for managers dealing with an invasive species. Specifically, for wild pigs, understanding the driving forces of resource selection at a fine scale can inform when, where, and how to deploy traps, toxicants, attractants, etc. to ensure visitations occur quickly and consistently^19,78^, as well as areas to focus mitigation efforts from wild pig damage. In addition, understanding how wild pigs use the landscape can provide an advantage for managers and/or disease biologists when trying to predict areas of high risk for disease transmission. Our results indicated vegetation class and other landscape features all determined habitat use by wild pigs when resting, foraging, and traveling. Therefore, targeting specific vegetation types, features, and times throughout the diel period could provide an advantage for managers when strategically employing specific management techniques in areas where wild pigs would be most vulnerable. For example, to increase efficiency and effectiveness of management techniques such as trapping and toxicant deployment, targeting wild pigs in habitat types they select for during the foraging and/or traveling behavioral states could greatly increase the number of pigs removed during these management processes^78^.

## Supplementary Information

Supplementary Information.

## Data Availability

The datasets used and/or analyzed during the current study are available from the corresponding author upon request.
