# Supplementary material for: Behavioral state resource selection in invasive wild pigs in the Southeastern United States
Source: Sci Rep. 2021 Mar 25;11:6924. doi: 10.1038/s41598-021-86363-3 (PMC7994638; doi:10.1038/s41598-021-86363-3)
Supplement: Supplementary file 1 — Supplementary Information. [file 41598_2021_86363_MOESM1_ESM.pdf]

Article title:

Behavioral State Resource Selection in Invasive Wild Pigs in the Southeastern United States

Journal name:

Scientific Reports

Author names:

Lindsay M. Clontz, Kim M. Pepin, Kurt C. VerCauteren, James C. Beasley

Affiliation and email address of corresponding author:

Savannah River Ecology Laboratory, Warnell School of Forestry and Natural Resources, University of Georgia, PO Drawer E, Aiken, SC 29802, USA

lindsay.clontz@uga.edu

Supplemental Table S1

Parameter	State 1	State 2	State 3
Avg. Step-length Minimum (m)	3.0	20.0	111.0
Avg. Step-length Maximum (m)	19.0	110.0	1500.0
Avg. Turn Angle (radians)	3.14	3.14	0.00

Supplemental Table S2

Analysis	Season		mean	sd	min	max
2nd Order RSF	Low-Forage	Distance to Stream	643.36	472.07	0.00	2670.67
		Distance to Primary Road	1573.54	1654.22	0.00	8669.38
		Distance to Secondary Road	146.82	164.75	0.00	2373.60
		Distance to Upland Hardwoods	206.16	198.73	0.00	2315.25
		Distance to Upland Pines	53.18	139.72	0.00	2296.52
		Distance to Shrub/Herb	207.11	266.24	0.00	2991.74
		Distance to Bottomland Hardwood	173.35	168.13	0.00	1259.29
		% Canopy Cover	69.95	29.54	0.00	100.00
2nd Order RSF	High-Forage	Distance to Stream	631.18	467.46	0.00	2618.43
		Distance to Primary Road	1634.48	1732.57	0.00	8668.29
		Distance to Secondary Road	145.60	161.55	0.00	2363.15
		Distance to Upland Hardwoods	204.78	197.48	0.00	2310.00
		Distance to Upland Pines	55.09	137.03	0.00	2303.76
		Distance to Shrub/Herb	207.49	262.16	0.00	2978.93
		Distance to Bottomland Hardwood	174.69	170.90	0.00	1288.60
		% Canopy Cover	69.33	30.05	0.00	100.00
3rd Order RSF	Low-Forage	Distance to Stream	576.04	482.41	0.00	2241.99
		Distance to Primary Road	1311.39	1414.48	0.00	7956.11
		Distance to Secondary Road	136.29	110.36	0.00	778.27
		Distance to Upland Hardwoods	187.87	159.81	0.00	1209.34
		Distance to Upland Pines	37.99	53.27	0.00	536.66
		Distance to Shrub/Herb	157.64	141.89	0.00	800.50
		Distance to Bottomland Hardwood	140.55	149.17	0.00	953.41
		% Canopy Cover	72.27	29.18	0.00	100.00
3rd Order RSF	High-Forage	Distance to Stream	596.38	484.95	0.00	2278.82
		Distance to Primary Road	1480.70	1615.76	0.00	7865.55
		Distance to Secondary Road	136.41	116.31	0.00	1140.39
		Distance to Upland Hardwoods	177.18	158.12	0.00	1273.50
		Distance to Upland Pines	40.01	66.07	0.00	1087.06
		Distance to Shrub/Herb	172.38	160.00	0.00	1766.95

	Distance to Bottomland Hardwood	146.99	155.12	0.00	1005.78
	% Canopy Cover	72.78	28.06	0.00	100.00

Supplemental Table S3

(a)

Rank	Model	ΔLogLik	ΔAIC	Weight
**1**	3 State: Hour	0.00	0.00	1.00
**2**	3 State: Null	202.31	380.61	0.00
**3**	2 State: Hour	1333.04	2632.08	0.00
**4**	2 State: Null	1501.39	2960.77	0.00

(b)

Rank	Model	ΔLogLik	ΔAIC	Weight
**1**	3 State: Hour	0.00	0.00	1.00
**2**	3 State: Null	211.36	398.72	0.00
**3**	2 State: Hour	1642.37	3250.73	0.00
**4**	2 State: Null	1747.69	3453.38	0.00

(c)

Rank	Model	ΔLogLik	ΔAIC	Weight
**1**	3 State: Hour	0.00	0.00	1.00
**2**	3 State: Null	827.61	1631.22	0.00
**3**	2 State: Hour	1823.26	3608.52	0.00
**4**	2 State: Null	1875.66	3709.33	0.00

(d)

Rank	Model	ΔLogLik	ΔAIC	Weight
**1**	3 State: Hour	0.00	0.00	1.00
**2**	3 State: Null	1400.02	2776.03	0.00
**3**	2 State: Hour	2442.18	4846.36	0.00
**4**	2 State: Null	2582.5	5122.99	0.00

Supplemental Table S4

Season	Sex	Variable	Estimate	Lower 95% CI	Upper 95% CI
Low-Forage	Female	Distance to upland hardwoods	-0.1749 ± 0.0102	-0.1950	-0.1550
		Distance to upland pines	-0.6287 ± 0.0225	-0.6730	-0.5848
		Distance to shrub/herb	-0.4026 ± 0.0131	-0.4286	-0.3771
		Distance to streams	-0.0911 ± 0.0082	-0.1071	-0.0752
		Distance to primary road	0.1573 ± 0.0077	0.1422	0.1723
		Distance to Secondary road	0.1879 ± 0.0118	0.1649	0.2110
		% Canopy cover	-0.0687 ± 0.0098	-0.0879	-0.0496
		Distance to bottomland hardwoods	-0.0896 ± 0.0088	-0.1068	-0.0724
Low-Forage	Male	Distance to upland hardwoods	-0.2649 ± 0.0106	-0.2857	-0.2441
		Distance to upland pines	-0.3284 ± 0.0204	-0.3685	-0.2886
		Distance to shrub/herb	-0.3846 ± 0.0131	-0.4103	-0.3590
		Distance to streams	-0.2546 ± 0.0088	-0.2718	-0.2374
		Distance to primary road	0.1055 ± 0.0082	0.0894	0.1215
		Distance to Secondary road	0.1047 ± 0.0119	0.0813	0.1281
		% Canopy cover	-0.0415 ± 0.0099	-0.0608	-0.0221
		Distance to bottomland hardwoods	-0.056 ± 0.0092	-0.0737	-0.0378
High-Forage	Female	Distance to upland hardwoods	0.0255 ± 0.0089	0.0080	0.0429
		Distance to upland pines	-0.3261 ± 0.0197	-0.3649	-0.2876
		Distance to shrub/herb	-0.5983 ± 0.0151	-0.6281	-0.5687
		Distance to streams	-0.2878 ± 0.0091	-0.3057	-0.2701
		Distance to primary road	-0.4510 ± 0.0109	-0.4725	-0.4296
		Distance to Secondary road	0.0577 ± 0.0123	0.0335	0.0819
		% Canopy cover	-0.0084 ± 0.0091	-0.0262	0.0095
		Distance to bottomland hardwoods	-0.0941 ± 0.0088	-0.1114	-0.0768
High-Forage	Male	Distance to upland hardwoods	0.0295 ± 0.0077	0.0145	0.0447
		Distance to upland pines	-0.1031 ± 0.0133	-0.1293	-0.0771
		Distance to shrub/herb	-0.3803 ± 0.0106	-0.4012	-0.3595
		Distance to streams	-0.1955 ± 0.0072	-0.2096	-0.1814
		Distance to primary road	-0.0034 ± 0.0075	-0.0181	0.0112
		Distance to Secondary road	0.0034 ± 0.0099	-0.0160	0.0228
		% Canopy cover	-0.055 ± 0.0078	-0.0704	-0.0397

			Distance to bottomland hardwoods	-0.044 ± 0.0073	-0.0578	-0.0293

Supplemental Table S5

Model	Season	Sex	Variable	Estimate ± SE	Lower 95% CI	Upper 95% CI
Resting (State 1)	Low-Forage	Female	Distance to upland hardwoods	0.0003 ± 0.0195	-0.0379	0.0385
			Distance to upland pines	-0.0240 ± 0.0185	-0.0601	0.0122
			Distance to shrub/herb	-0.1462 ± 0.0184	-0.1823	-0.1102
			Distance to stream	0.0870 ± 0.0171	0.0535	0.1204
			Distance to primary road	-0.0586 ± 0.0193	-0.0964	-0.0208
			Distance to secondary road	0.0807 ± 0.0166	0.0481	0.1133
			% canopy cover	0.1693 ± 0.0185	0.1330	0.2057
			Distance to bottomland hardwoods	-0.3808 ± 0.0187	-0.4174	-0.3442
Foraging (State 2)	Low-Forage	Female	Distance to upland hardwoods	0.0693 ± 0.0188	0.0324	0.1062
			Distance to upland pines	0.0182 ± 0.0197	-0.0205	0.0568
			Distance to shrub/herb	0.0319 ± 0.0157	0.0012	0.0627
			Distance to stream	0.0075 ± 0.0161	-0.0240	0.0390
			Distance to primary road	-0.2293 ± 0.0173	-0.2631	-0.1954
			Distance to secondary road	0.1693 ± 0.0148	0.1402	0.1984
			% canopy cover	0.7363 ± 0.0261	0.6852	0.7874
			Distance to bottomland hardwoods	-0.5683 ± 0.0213	-0.6099	-0.5266
Traveling (State 3)	Low-Forage	Female	Distance to upland hardwoods	0.0224 ± 0.0152	-0.0075	0.0523
			Distance to upland pines	0.1283 ± 0.0145	0.0999	0.1567
			Distance to shrub/herb	0.0694 ± 0.0132	0.0434	0.0953
			Distance to stream	-0.0205 ± 0.0139	-0.0477	0.0067
			Distance to primary road	-0.0712 ± 0.0136	-0.0979	-0.0445
			Distance to secondary road	0.0007 ± 0.0125	-0.0237	0.0251
			% canopy cover	0.2479 ± 0.0164	0.2156	0.2801

			Distance to bottomland hardwoods	-0.4449 ± 0.0158	-0.4759	-0.4139
Resting (State 1)	Low-Forage	Male	Distance to upland hardwoods	-0.1216 ± 0.0165	-0.1540	-0.0893
			Distance to upland pines	0.2687 ± 0.0165	0.2364	0.3011
			Distance to shrub/herb	-0.1592 ± 0.0218	-0.2018	-0.1165
			Distance to stream	-0.2701 ± 0.0248	-0.3188	-0.2214
			Distance to primary road	-0.4467 ± 0.0413	-0.5277	-0.3657
			Distance to secondary road	0.2468 ± 0.0177	0.2121	0.2815
			% canopy cover	0.0697 ± 0.0187	0.0331	0.1063
			Distance to bottomland hardwoods	-0.2738 ± 0.0221	-0.3171	-0.2305
Foraging (State 2)	Low-Forage	Male	Distance to upland hardwoods	-0.0008 ± 0.0185	-0.0370	0.0353
			Distance to upland pines	0.1496 ± 0.0194	0.1117	0.1876
			Distance to shrub/herb	-0.053 ± 0.0210	-0.0943	-0.0120
			Distance to stream	-0.004 ± 0.0240	-0.0514	0.0426
			Distance to primary road	-0.2104 ± 0.0389	-0.2866	-0.1342
			Distance to secondary road	-0.0393 ± 0.0192	-0.0769	-0.0017
			% canopy cover	0.7822 ± 0.0322	0.7192	0.8453
			Distance to bottomland hardwoods	-0.4846 ± 0.0255	-0.5345	-0.4347
Traveling (State 3)	Low-Forage	Male	Distance to upland hardwoods	0.0498 ± 0.0130	0.0244	0.0752
			Distance to upland pines	0.1250 ± 0.0150	0.0955	0.1544
			Distance to shrub/herb	-0.1626 ± 0.0172	-0.1962	-0.1289
			Distance to stream	0.0292 ± 0.0196	-0.0093	0.0676
			Distance to primary road	-0.1294 ± 0.0308	-0.1897	-0.0691
			Distance to secondary road	-0.1961 ± 0.0160	-0.2275	-0.1647
			% canopy cover	0.2095 ± 0.0168	0.1765	0.2425
			Distance to bottomland hardwoods	-0.3702 ± 0.0179	-0.4053	-0.3351

Resting (State 1)	High-Forage	Female	Distance to upland hardwoods	-0.3794 ± 0.1907	-0.7532	-0.0057
			Distance to upland pines	-0.2719 ± 0.0148	-0.3009	-0.2429
			Distance to shrub/herb	-0.0053 ± 0.0138	-0.0325	0.0218
			Distance to stream	-0.1325 ± 0.0145	-0.1609	-0.1042
			Distance to primary road	0.2204 ± 0.0154	0.1903	0.2505
			Distance to secondary road	-0.1956 ± 0.0122	-0.2195	-0.1717
			% canopy cover	0.0721 ± 0.0128	0.0470	0.0972
			Distance to bottomland hardwoods	0.4200 ± 0.0161	0.3885	0.4514
Foraging (State 2)	High-Forage	Female	Distance to upland hardwoods	-0.3463 ± 0.0151	-0.3758	-0.3167
			Distance to upland pines	-0.0749 ± 0.0170	-0.1081	-0.0416
			Distance to shrub/herb	0.0317 ± 0.0168	-0.0012	0.0646
			Distance to stream	0.1333 ± 0.0195	0.0950	0.1715
			Distance to primary road	-0.3230 ± 0.0156	-0.3536	-0.2924
			Distance to secondary road	0.0643 ± 0.0157	0.0335	0.0951
			% canopy cover	0.5632 ± 0.0233	0.5175	0.6089
			Distance to bottomland hardwoods	-0.2882 ± 0.0197	-0.3268	-0.2496
Traveling (State 3)	High-Forage	Female	Distance to upland hardwoods	-0.1999 ± 0.0134	-0.2262	-0.1737
			Distance to upland pines	-0.0271 ± 0.0129	-0.0523	-0.0018
			Distance to shrub/herb	0.0104 ± 0.0131	-0.0153	0.0361
			Distance to stream	0.1528 ± 0.0151	0.1233	0.1823
			Distance to primary road	-0.1890 ± 0.0114	-0.2114	-0.1667
			Distance to secondary road	0.0648 ± 0.0115	0.0423	0.0872
			% canopy cover	0.2767 ± 0.0141	0.2491	0.3044
			Distance to bottomland hardwoods	-0.2710 ± 0.0139	-0.2982	-0.2438
Resting (State 1)	High-Forage	Male	Distance to upland hardwoods	-0.1152 ± 0.0144	-0.1434	-0.0871
			Distance to upland pines	0.2054 ± 0.0162	0.1737	0.2372

			Distance to shrub/herb	-0.2508 ± 0.0190	-0.2880	-0.2135
			Distance to stream	0.0266 ± 0.0188	-0.0103	0.0634
			Distance to primary road	-0.2176 ± 0.0314	-0.2791	-0.1561
			Distance to secondary road	0.1562 ± 0.0158	0.1252	0.1872
			% canopy cover	0.3785 ± 0.0175	0.3442	0.4128
			Distance to bottomland hardwoods	-0.2933 ± 0.0173	-0.3272	-0.2595
Foraging (State 2)	High-Forage	Male	Distance to upland hardwoods	0.0963 ± 0.0159	0.0652	0.1275
			Distance to upland pines	0.1670 ± 0.0185	0.1308	0.2033
			Distance to shrub/herb	-0.018 ± 0.0183	-0.0542	0.0173
			Distance to stream	0.1050 ± 0.0205	0.0649	0.1451
			Distance to primary road	-0.0203 ± 0.0309	-0.0808	0.0401
			Distance to secondary road	0.0516 ± 0.0167	0.0188	0.0843
			% canopy cover	0.6411 ± 0.0254	0.5914	0.6908
			Distance to bottomland hardwoods	-0.4063 ± 0.0209	-0.4473	-0.3652
Traveling (State 3)	Low-Forage	Male	Distance to upland hardwoods	-0.0145 ± 0.0109	-0.0358	0.0068
			Distance to upland pines	0.1127 ± 0.0127	0.0878	0.1375
			Distance to shrub/herb	-0.1358 ± 0.0138	-0.1629	-0.1087
			Distance to stream	0.1466 ± 0.0148	0.1176	0.1756
			Distance to primary road	-0.0373 ± 0.0229	-0.0822	0.0075
			Distance to secondary road	-0.1980 ± 0.0129	-0.2232	-0.1727
			% canopy cover	0.1758 ± 0.0125	0.1512	0.2003
			Distance to bottomland hardwoods	-0.1890 ± 0.0124	-0.2133	-0.1647
